# An R2R3-MYB Transcription Factor Positively Regulates the Glandular Secretory Trichome Initiation in *Artemisia annua* L.

**DOI:** 10.3389/fpls.2021.657156

**Published:** 2021-04-09

**Authors:** Wei Qin, Lihui Xie, Yongpeng Li, Hang Liu, Xueqing Fu, Tiantian Chen, Danial Hassani, Ling Li, Xiaofen Sun, Kexuan Tang

**Affiliations:** Joint International Research Laboratory of Metabolic and Developmental Sciences, Key Laboratory of Urban Agriculture (South) Ministry of Agriculture, Plant Biotechnology Research Center, Fudan-SJTU-Nottingham Plant Biotechnology R&D Center, School of Agriculture and Biology, Shanghai Jiao Tong University, Shanghai, China

**Keywords:** *Artemisia annua* L., trichome initiation, transcription factor, glandular trichome, R2R3 MYB

## Abstract

*Artemisia annua* L. is known for its specific product “artemisinin” which is an active ingredient for curing malaria. Artemisinin is secreted and accumulated in the glandular secretory trichomes (GSTs) on *A. annua* leaves. Earlier studies have shown that increasing GST density is effective in increasing artemisinin content. However, the mechanism of GST initiation is not fully understood. To this end, we isolated and characterized an R2R3-MYB gene, *AaMYB17*, which is expressed specifically in the GSTs of shoot tips. Overexpression of *AaMYB17* in *A. annua* increased GST density and enhanced the artemisinin content, whereas RNA interference of *AaMYB17* resulted in the reduction of GST density and artemisinin content. Additionally, neither overexpression lines nor RNAi lines showed an abnormal phenotype in plant growth and the morphology of GSTs. Our study demonstrates that AaMYB17 is a positive regulator of GSTs’ initiation, without influencing the trichome morphology.

## Introduction

Malaria is a mosquito-borne infectious disease caused by the *Plasmodium* species which targets human red blood cells (Wilson et al., 2011). According to the World Health Organization (WHO), malaria is responsible for 228 million cases, including 405,000 deaths worldwide in 2018 (World Health Organization [WHO], 2019). Artemisinin-based combination therapy (ACT) is considered the most efficient treatment to cure malaria (World Health Organization [WHO], 2019). Artemisinin is a bioactive compound, synthesized and accumulated in the glandular trichome of a traditional Chinese herb, *Artemisia annua* L. (Graham et al., 2010; Tan et al., 2015).

Trichomes are unicellular or multicellular structures derived from aerial epidermal cells (Duke and Paul, 2013). Their morphological diversity, different number of cells, and their potential in secondary metabolism distinguish them from each other (Werker, 2000; Serna and Martin, 2006). Depending on secondary metabolism capability, they are divided into glandular trichomes and non-glandular trichomes (Huchelmann et al., 2017). The powerful biosynthetic secreting and accumulating capabilities of glandular trichomes for the production of plants’ secondary metabolites including terpenoids, flavonoid, alkaloids, polysaccharides, polyphenols, and phenylpropanoids have made them an exciting research topic in recent years for plant metabolic engineering strategies (Wagner et al., 2004; Fridman et al., 2005; Gershenzon and Dudareva, 2007; Xie et al., 2008; Tattini et al., 2010; Weinhold and Baldwin, 2011). Many efforts have been dedicated to promoting the accumulation of secondary metabolites in plants, and it is feasible to improve secondary metabolic productivity by increasing glandular trichome density (Tissier, 2012). Unlike glandular trichomes, non-glandular trichomes are not efficient in secondary metabolism (Soetaert et al., 2013; Huchelmann et al., 2017). Besides, both non-glandular and glandular trichomes play an essential role in the defense against abiotic and biotic stress, such as UV light, water absorption, salt stress, and herbivores (Mauricio and Rausher, 1997; Serna and Martin, 2006). Therefore, systematic investigations on the molecular basis of trichome initiation are needed.

There are two types of trichomes on the surface of *A. annua* leaves, glandular secretory trichomes (GSTs) and T-shaped trichomes (TSTs), both have multicellular structures (Duke and Paul, 2013; Xiao et al., 2016). Artemisinin is specifically synthesized and accumulated in GSTs. Thus, promoting GST initiation is a potential strategy for increasing the artemisinin content in *A. annua*. Even though the mechanism of unicellular trichome initiation in Arabidopsis has been studied thoroughly, the mechanism of multicellular trichome initiation might vary and needs further investigation (Payne et al., 1999; Martin, 2006; Maes et al., 2011; Yang et al., 2011).

In plants, transcription factors regulate a variety of biological processes (Mitsuda and Ohme-Takagi, 2009). Many TF families including the well-known MYB family were reported to regulate trichome initiation. MYBs are a family of proteins that contain the conserved MYB DNA-binding domain (Romero et al., 1998). MYB proteins are classified into four classes according to the number of adjacent repeats (one, two, three, or four): 4R-MYB, R1R2R3-type MYB (3R-MYB), 1R-MYB/MYB-related, and R2R3-MYB (Rosinski and Atchley, 1998; Jin and Martin, 1999; Stracke et al., 2001; Dubos et al., 2010). The R2R3-MYBs are the largest subfamily of MYB TFs containing an N terminal DNA-binding domain and a C terminus activation or repression domain (Martin and Paz-Ares, 1997; Dubos et al., 2010). In recent studies, the R2R3-MYBs have been proven to regulate primary and secondary metabolism, plant development, cell fate and identity, response to biotic and abiotic stresses, and light and hormone signaling pathways (Martin and Paz-Ares, 1997; Dubos et al., 2010; Brockington et al., 2013). Based on the conserved amino-acid sequence motifs present at the carboxy terminal to the MYB domain, R2R3-type MYB factors have been categorized into 22 subgroups (Martin and Paz-Ares, 1997; Kranz et al., 2010). Former studies have reported that subgroup 9 of MYB TFs, including MIXTA or MIXTA-like, are essential in plant cellular differentiation, especially in epidermal cells differentiation (Brockington et al., 2013). For instance, the first MIXTA characterized is the snapdragon *Antirrhinum majus* MIXTA (AmMIXTA) which controls formation of the conical shape of petal epidermal cells (Noda et al., 1994). Moreover, some other *MIXTA* genes in *A. majus*, such as *AmMYBML1*, are also described as having a similar function (Oshima et al., 2013). *AtMYB106* and *AtMYB16* which also belong to subgroup 9 regulate trichome branch formation and epidermal cell morphology in Arabidopsis (Jakoby et al., 2008; Oshima et al., 2013). Furthermore, *AaMIXTA1*, belonging to subgroup 9, positively regulates glandular trichome initiation and cuticle biosynthesis in *A. annua* (Shi et al., 2018). In *Populus euphratica*, overexpression of an *AtMYB106* homologous gene, *PtaMYB186*, can increase trichome density (Plett et al., 2010). Nevertheless, the molecular mechanism of MYB-involved trichome initiation needs further study.

In this study, an R2R3-MYB gene, *AaMYB17*, was isolated from 13 MYBs which are highly and specifically expressed in the meristem. Overexpression of *AaMYB17* increased the GST density, whereas RNA interference of *AaMYB17* resulted in the decrease of GST density in *A. annua*. Taken together, we identified a new TF which could promote artemisinin accumulation by positively regulating the GST density in *A. annua* leaves.

## Materials and Methods

### Plant Materials and Growth Conditions

*The A. annua* we used in this study was “Huhao 1,” which originated from Chongqing, China and has been planted and selected in Shanghai, China for several years (Shen et al., 2016). The plants were grown under a 16 h/8 h light/dark photoperiod and 65% relative humidity at 25 ± 2°C. *Nicotiana benthamiana*, used for transient transformation, was grown at 25 ± 2°C under a 16-h light photoperiod.

### Phylogenetic Tree and Amino Acid Sequence Alignment

Myeloblastosis (MYB) TFs highly and specifically expressed in the meristem were identified from our unpublished transcriptome data (Shi et al., 2018). The phylogenetic tree was analyzed by Mega5 software (Tamura et al., 2011). Protein sequences of MIXTA or MIXTA-like TFs from other species were downloaded from the National Center for Biotechnology Information (NCBI). Amino acid sequence alignment of AaMYB17 and MIXTA/MIXTA-like proteins from other species was performed with Genedoc (Nicholas, 1997).

### RNA Isolation and Reverse Transcription

RNA of different tissues and leaves from different phyllotaxis of *A. annua* was extracted using the RNA prep Pure Plant Kit following the manufacturer’s instructions (Tiangen, Beijing, China). Different tissues (flower bud, leaves, flower, stem, and root) of the wild-type plants were collected from 6-month-old *A. annua* grown in a glasshouse. In addition, the leaf samples were gathered from leaf 0 (meristem), leaf 1 (first leaf below meristem), leaf 2, leaf 3, leaf 4, leaf 5, leaf 9, and leaf 16, counting from the apical top of the main stem (Yan et al., 2016). RNA samples were reverse transcribed into cDNA using the PrimeScript II RT Master Mix (Takara, Dalian, China).

### Quantitative Real-Time PCR (qRT-PCR)

Quantitative real-time PCR was performed on a Roche LightCycler 96 real-time PCR machine (Roche, Basel, Switzerland) using SuperReal PreMix Plus SYBR-Green (Tiangen Biotech, China). qRT-PCR was conducted as described previously (Shen et al., 2016), and the relative expression levels were calculated as described previously (Livak and Schmittgen, 2002). The experiments were performed using three biological replicates. All the primers used in the qRT-PCR are listed in Supplementary Table 1.

### Transformation of *A. annua*

The full-length cDNA sequence of *AaMYB17* was amplified using the cDNA of the *A. annua* meristem through PCR using KOD plus DNA polymerase (Toyobo, Osaka, Japan). It was further cloned into a pHB vector under a double CaMV35S promoter to generate pHB-CaMV35S:*AaMYB17*-YFP:NOS with the YFP fused to the C-terminal of *AaMYB17* (Mao et al., 2005). A 269 bp *AaMYB17* fragment was recombined into the phellsgate12 vector via a gateway LR recombination reaction (Invitrogen) to construct the *AaMYB17*-RNAi vector. A 2185 bp *AaMYB17* promoter fragment was cloned for the construction of pCAMBIA 1391Z-PMYB17. These constructs were introduced into *Agrobacterium tumefaciens* strain EHA105, following *Agrobacterium*-mediated transformation of *A. annua* as described previously (Shen et al., 2016).

### Subcellular Localization of AaMYB17

The full-length ORF of the *AaMYB17* gene without the terminator codon was inserted into the pHB-YFP expression vector under the CaMV35S promoter to form a pHB-AaMYB17–YFP fusion protein. Then the plasmid and p19 protein were introduced into the *A. tumefaciens* strain GV3101 for *N. benthamiana* leaf transient expression (Sparkes et al., 2006). The fluorescent signals were observed 60–72 h after infiltration using a TCS SP5-II confocal laser microscopy (Leica Microsystems, Wetzlar, Germany). Three biological repeats were performed to verify these results.

### GUS Staining Assay

A 2185 bp *AaMYB17* promoter fragment was cloned using KODFX (Toyobo, Japan) and inserted into the pCAMBIA1391Z vector which carries the GUS gene. This construction was transformed into *A. annua*. The GUS assay of the transgenic plants (T1) was performed as previously described (Jefferson et al., 1987). Transgenic plants were stained in a GUS staining solution and incubated at 37°C in the dark overnight. After GUS staining, ethyl alcohol was used to remove chlorophyll.

### Glandular Trichome Density Counting

The mature leaves (leaf 9, the ninth leaf below the meristem) of *A. annua* plants (T0) grown in the glasshouse were selected to count the density of glandular trichomes. Each leaf was imaged by a ×5 objective using fluorescence microscopy (Olympus, Tokyo, Japan). The ImageJ program^1^ was used to measure the leaf area and the number of glandular trichomes, as previously described (Cheng et al., 2014). Three different leaves of each independent plant at the same position were selected to count the trichome numbers.

### Scanning Electron Microscopy (SEM)

Mature leaves (leaf 9, the ninth leaf below the meristem) of *A. annua* plants (T0) grown in the glasshouse were selected and treated following the previously reported method (Singh et al., 2016). Leaves were imaged with a Hitachi (Hitachi Ltd., Tokyo, Japan) S-3400N scanning electron microscope.

### Artemisinin Content Measurement

Leaves of 5-month-old *A. annua* plants (T0) grown in the glasshouse were gathered and dried in 50°C for 24 h to measure the artemisinin content. The dried leaves were ground into powder samples, and 1 g of each sample was extracted by methanol and treated ultrasonically twice (55 HZ, 30 min). The artemisinin content was measured by the Waters Alliance 2695 HPLC system (Milford, MA, United States) using high-performance liquid chromatography (HPLC) as described previously (Shen et al., 2016). Three biological repeats were measured for each sample.

### Dual-Luciferase (Dual-LUC) Assay

PHB-*AaMYB17* was transformed into *A. tumefaciens* strain GV3101 to act as an effector. The empty pHB vector was used as a control. The promoters of *ADS*, *CYP71AV1*, *DBR2*, *ALDH1*, *AaKCS5*, *AaCER1*, *AaCYP77A1*, *AaCYP86A1*, and *AaABCG12* were cloned into the vector pGREEN II0800 to act as the reporters. GV3101 strains harboring the indicated combinations of effectors and reporters were co-infiltrated into 6-week-old *N. benthamiana* leaves. The leaves of *N. benthamiana* were gathered after 24 h of cultivation in dark conditions and 24 h of cultivation in light conditions. After being quick-frozen in liquid nitrogen, the samples were subjected to firefly LUC and REN activities analysis using the Dual-Luciferase^®^ Reporter Assay System (Promega, United States). Three biological repeats were measured for each sample.

## Results

### Identification and Characterization of *AaMYB17*

**FIGURE 1:**
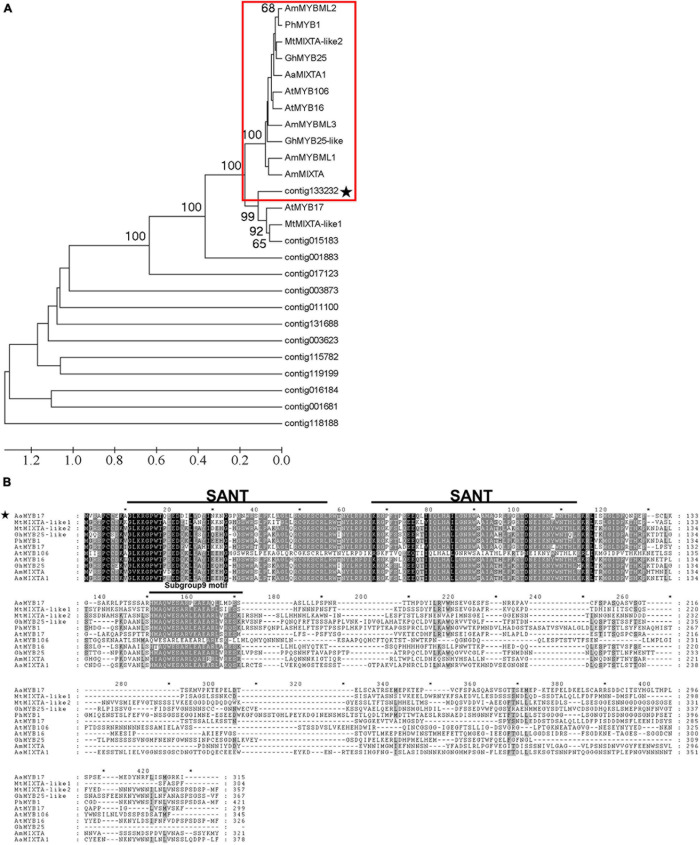
Identification and characterization of *AaMYB17*. **(A)** Phylogenetic tree of MYB proteins expressed in young leaf trichomes, flower bud trichomes, and shoots of *A. annua*, and some MYBs involved in organ development and cell differentiation from other species. The subgroup 9 MYBs described previously are highlighted in red, and the candidate contig is starred. Sequences were downloaded from the TAIR and NCBI databases. **(B)** Amino acid sequence alignment of AaMYB17 and MIXTA/MIXTA-like proteins from other species were performed with Genedoc (Nicholas, 1997). The conserved MYB domain and subgroup 9 motif are represented by a line above the sequence. The candidate is starred.

To further study the function of MYB proteins in trichome initiation, we selected 13 MYBs which are highly and specifically expressed in the meristem where trichome initiation occurs using the GST transcriptome database (Shi et al., 2018) and other published databases (Graham et al., 2010). Phylogenetic analysis of these 13 candidate MYBs revealed that contig133232 clustered with the MIXTA and MIXTA-like TFs from the other species (Figure 1A). Moreover, contig133232 was found to share a highly conserved R2R3-MYB domain by means of an amino acid alignment (Figure 1B). The characteristic of “HMAQWESARxEAEAxLxMDS” demonstrated that contig133232 belonged to subgroup 9 of R2R3-MYBs (Stracke et al., 2001; Brockington et al., 2013). We further named contig133232 as *AaMYB17* which is homologous with *AtMYB17* in *Arabidopsis thaliana* and selected it as a candidate for further study.

### Expression Profile of *AaMYB17*

**FIGURE 2:**
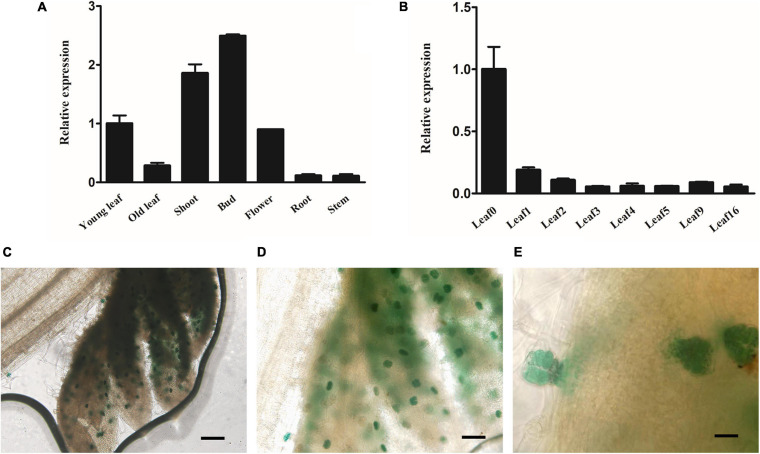
Expression profile of *AaMYB17*. **(A)** Relative expression of *AaMYB17* in young leaf, old leaf, shoot, bud, flower, root, and stem of *A. annua* by quantitative real-time polymerase chain reaction. **(B)** Relative expression of *AaMYB17* in *A. annua* leaves of different developmental ages. β-actin was used as an internal control in **A** and **B**. Error bars represent the standard deviation (*n* = 3). **(C–E)** GUS staining of transgenic *A. annua* plants was observed in the GSTs of shoot tip. Bars: **(C)** 100 μm; **(D)** 50 μm, and **(E)** 20 μm.

qRT-PCR was performed using cDNA from different tissues to analyze the transcript level of *AaMYB17* and investigate its expression pattern. The results indicated that *AaMYB17* was highly expressed in the young leaf, bud, flower, and especially in the shoot, where GSTs were abundant (Figure 2A). The expression of *AaMYB17* in different leaves followed a rapid descending pattern associated with leaf aging (Figure 2B). To further explore the tissue-specific expression pattern of *AaMYB17*, we cloned a 2186-bp sequence of the *AaMYB17* promoter to generate a p*AaMYB17*-GUS plasmid, and transformed it into *A. annua*. GUS staining of the transgenic plants showed that *AaMYB17* was specifically expressed in shoot tip GSTs (Figures 2C–E and Supplementary Figure 1).

### Subcellular Localization of AaMYB17

**FIGURE 3:**
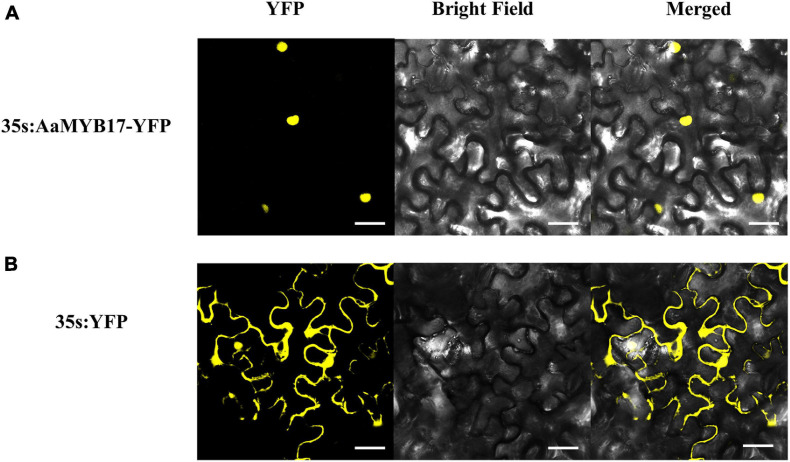
The subcellular localization of AaMYB17 in leaves of *N. benthamiana*. **(A)** Localization of AaMYB17 protein fused with YFP. **(B)** Localization of empty vector. Bars = 40 μm.

To investigate the subcellular localization of AaMYB17, a yellow fluorescent protein (YFP) was fused to the N-terminus of AaMYB17. The YFP fluorescence of 35s: AaMYB17-YFP was observed in the nucleus of *N. benthamiana* epidermal cells (Figure 3A). However, the fluorescence of control was observed from the whole cell of *N. benthamiana* (Figure 3B). These results demonstrated that AaMYB17 was localized in the nucleus, which is also consistent with its role as a TF.

### AaMYB17 Positively Regulates GST Initiation in *A. annua*

**FIGURE 4:**
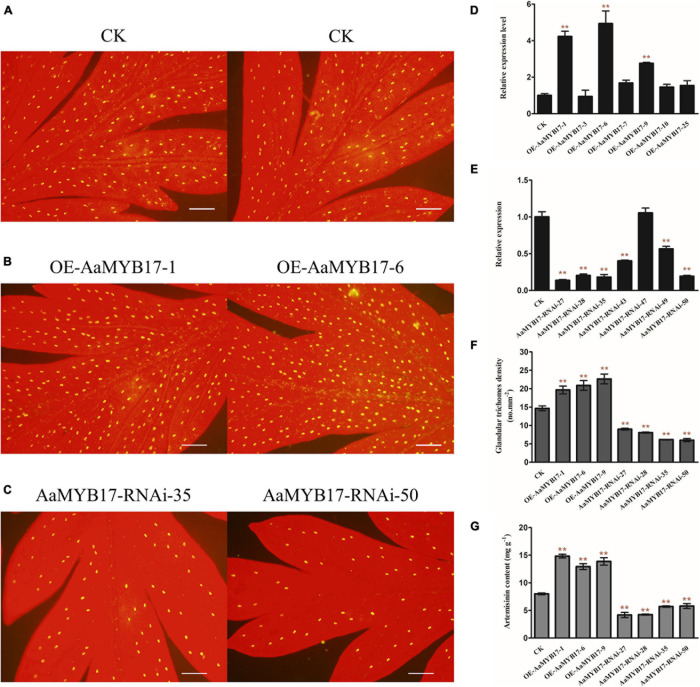
The expression of *AaMYB17* affects *A. annua* glandular trichome initiation. The glandular trichomes on the adaxial side of mature leaves derived from **(A)** wild-type (CK) plants, **(B)** *OE*-*AaMYB17* transgenic *A. annua* plants, and **(C)** *AaMYB17-RNAi* transgenic *A. annua* plants (bars, 200 μm). Expression level of *AaMYB17* in the *A. annua* leaves in **(D)** *OE-AaMYB17* transgenic *A. annua* plants and **(E)** *AaMYB17*-*RNAi* transgenic *A. annua* plants. **(F)** Glandular trichomes density of mature leaves derived from CK and *AaMYB17* transgenic plants. **(G)** High-performance liquid chromatography (HPLC) analysis of the artemisinin content (mg g^–1^ leaf DW) in transgenic *A. annua* plants. Data are given as means ± SD (*n* = 3) (**P < 0.01; Dunnett’s test).

**FIGURE 5:**
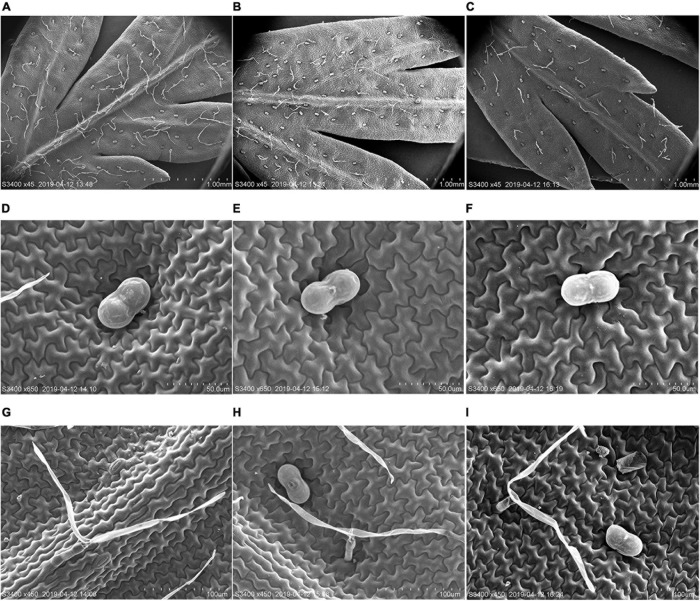
Scanning electron microscope (SEM) analysis of the surface of leaves. **(A)** GSTs and T-shape trichomes (TSTs) on the adaxial sides of mature leaves derived from wild-type plants, **(B)** *OE-AaMYB17* transgenic *A. annua* plants, and **(C)** *AaMYB17-RNAi* transgenic *A. annua* plants. **(D)** The morphologies of GST on the leaf of wild-type plants, **(E)** *OE-AaMYB17* transgenic *A. annua* plants, and **(F)** *AaMYB17-RNAi* transgenic *A. annua* plants. **(G)** The morphologies of TST on the leaf of wild-type plants, **(H)** *OE-AaMYB17* transgenic *A. annua* plants, and **(I)** *AaMYB17-RNAi* transgenic *A. annua* plants. Bars: **(A–C)** 1.00 mm; **(D–F)** 50 μm; and **(G–I)** 100 μm.

To further clarify the molecular function of *AaMYB17* in *A. annua*, we generated *AaMYB17*-overexpressed transgenic plants driven by the cauliflower mosaic virus (CaMV) 35S promoter. Quantitative RT-PCR was performed and revealed that *AaMYB17* was significantly overexpressed in transgenic plants (Figure 4D). In three independent overexpression lines, the number of GSTs on the adaxial leaf side was increased 1.3–1.6-fold compared with the check control (Figures 4A,B,F). As expected, the artemisinin content of the *OE*-*AaMYB17* lines was increased from 8 to 15 mg g^–1^ DW compared to the wild-type plants (Figure 4G). On the other hand, *AaMYB17*-suppressed RNAi lines were generated under the CaMV 35S promoter and *AaMYB17* expression decreased significantly (Figure 4E). In these four independent RNAi lines, the number of GSTs on the adaxial leaf side was decreased 1.5- to 2.3-fold compared with check control (Figures 4A,C,F). The artemisinin content of the *AaMYB17-RNAi* lines was decreased from 8 to 6 mg g^–1^ DW compared to the control (Figure 4G). Meanwhile, the shape of GSTs and TSTs was observed using SEM, and there was no difference between transgenic plants and wild-type plants (Figures 5A–I). Furthermore, neither the overexpression nor the RNAi of *AaMYB17* affected the growth of transgenic plants (Supplementary Figure 2).

The dual-LUC assay was performed to detect whether AaMYB17 activated the expression of the key enzyme genes in the artemisinin biosynthesis pathway, such as *ADS*, *CYP71AV1*, *DBR2*, and *ALDH1*. It showed that AaMYB17 had no significant influence on them (Supplementary Figure 3). These results indicated that AaMYB17 regulated trichome initiation positively.

## Discussion

Former studies revealed that MIXTA/MIXTA-like TFs play an important role in the regulation of trichome initiation or cell development. *A. majus MIXTA* (*AmMIXTA*) as the first *MIXTA* gene to be reported regulates the development and initiation of the conical cell shape of the petal epidermis (Noda et al., 1994; Glover et al., 1998). *AtMYB16* and *AtMYB106* of *A. thaliana* are also reported to regulate trichome development (Baumann et al., 2007; Jakoby et al., 2008). In *Gossypium hirsuta*, *GhMYB25* and *GhMYB25-like* genes regulate early cotton fiber and trichome development (Machado et al., 2009; Walford et al., 2011). These studies demonstrate that *MIXTA/MIXTA-like* genes play very crucial roles in trichome development and initiation. In this study, we characterized MIXTA TF AaMYB17. By generating overexpression transgenic *A. annua* plants, we found that AaMYB17 could increase GST density and artemisinin content compared to the control (Figures 4A,B,F,G). On the contrary, GST density and artemisinin content decreased in RNAi transgenic plants (Figures 4A,C,F,G).

Glandular trichomes are special structures derived from the epidermal cells of many plants (Werker, 2000). They have the potential to be a powerful secondary metabolic factory (Tissier, 2012). Furthermore, the various compounds in glandular trichomes are potential active ingredients for many applications, including curing diseases, in fragrance, in killing pests, etc. (Dixon, 2001; Schilmiller et al., 2008; Tissier, 2012; Huchelmann et al., 2017). In *A. annua*, one of the most well-known compounds is artemisinin which is synthesized in GSTs (Olofsson et al., 2011). It is feasible that artemisinin content could be increased by increasing GST density.

From the previous studies, R2R3-MYB and HD-ZIP IV TFs play very important roles in glandular trichome initiation in *A. annua*. AaMYB1 is the first R2R3-MYB found to regulate GST initiation positively in *A. annua* (Matías-Hernández et al., 2017). Moreover, AaMIXTA1 is found as a positive regulator in GST initiation (Shi et al., 2018). As AaMYB17 is homologous with AaMIXTA1, we tried to determine whether AaMYB17 and AaMIXTA1 have the same functions. qRT-PCR was performed to analyze the expression level of cutin- and wax-related synthase genes *AaCYP77A1*, *AaCYP86A1*, *AaABCG12*, *AaKCS5*, and *AaCER1*, which are activated by AaMIXTA1 significantly (Shi et al., 2018) in *AaMYB17* overexpression and RNAi transgenic *A. annua* plants. The results indicated that there were no apparent differences (Supplementary Figure 4). The dual-LUC assay results in *N. benthamiana* revealed that AaMYB17 had no significant influence on *AaCYP77A1*, *AaCYP86A1*, *AaABCG12*, *AaKCS5*, and *AaCER1* (Supplementary Figure 5). These results indicate that AaMYB17 differs from AaMIXTA1 in cuticle biosynthesis. Furthermore, two HD-ZIP IV TFs, AaHD1 and AaHD8, were found to positively regulate GST initiation (Yan et al., 2016, 2018). These studies indicate that R2R3-MYB and HD-ZIP IV TFs have a powerful function in GST initiation, and there may be more unknown R2R3-MYB and HD-ZIP IV TFs involved in GST initiation.

In *A. thaliana*, there is only one type of trichome which is non-glandular and unicellular. It is thought that unicellular trichomes and multicellular trichomes form a distinct pathway (Yang and Ye, 2013). In *A. annua*, there are two types of multicellular trichomes, GSTs and TSTs. This might provide a reference for multicellular trichome development. In this study, an R2R3-MYB transcription factor, AaMYB17, which positively regulates GST initiation, was identified. Our study expands the knowledge of the molecular mechanism of multicellular trichome initiation.

## Accession Numbers

AaMYB17: MW468051, AmMIXTA: CAA55725.1, AmMYBM L1: CAB43399.1, AmMYBML2: AAV70655.1, AmMYBML3: AAU13905.1, PhMYB1: CAA78386.1, AtMYB16: ANM690 24.1, AtMYB17: AEE80179.1, AtMYB106: NP_001326423.1, MtMIXTA-like1: XP_003623894.1, MtMIXTA-like2: XP_0036 18530.1, GhMYB25: AAK19616.1, and GhMYB25-like: ADZ98881.1.

## Data Availability Statement

The original contributions presented in the study are included in the article/Supplementary Material, further inquiries can be directed to the corresponding author.

## Author Contributions

WQ and KT designed the research. WQ, LX, YL, HL, and TC carried out the expression analysis, vector construction, transgenic plant generation, subcellular localization, SEM, and dual-luciferase. WQ drafted the manuscript. DH, YL, LX, LL, XS, and KT revised the manuscript. All authors approved the manuscript.

## Conflict of Interest

The authors declare that the research was conducted in the absence of any commercial or financial relationships that could be construed as a potential conflict of interest.

## Footnotes

1 http://rsb.info.nih.gov/ij
