# Supplementary material for: An R2R3-MYB Transcription Factor Positively Regulates the Glandular Secretory Trichome Initiation in Artemisia annua L
Source: Front Plant Sci. 2021 Apr 9;12:657156. doi: 10.3389/fpls.2021.657156 (PMC8063117; doi:10.3389/fpls.2021.657156)
Supplement: Supplementary file 1 [file Data_Sheet_1.docx]

***Supplementary Material***

**An R2R3-MYB transcription factor positively regulates the glandular secretory trichome initiation in *Artemisia annua* L*.***

**Wei Qin, Lihui Xie, Yongpeng Li, Hang Liu, Xueqing Fu, Tiantian Chen, Danial Hassani, Ling Li, Xiaofen Sun and Kexuan Tang***

*** Correspondence:**Kexuan Tang
[kxtang@sjtu.edu.cn](mailto:kxtang@sjtu.edu.cn)


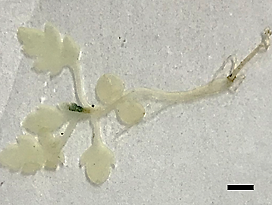


Figure. S1: GUS staining of total transgenic *A. annua* plants. Bar:0.3 cm


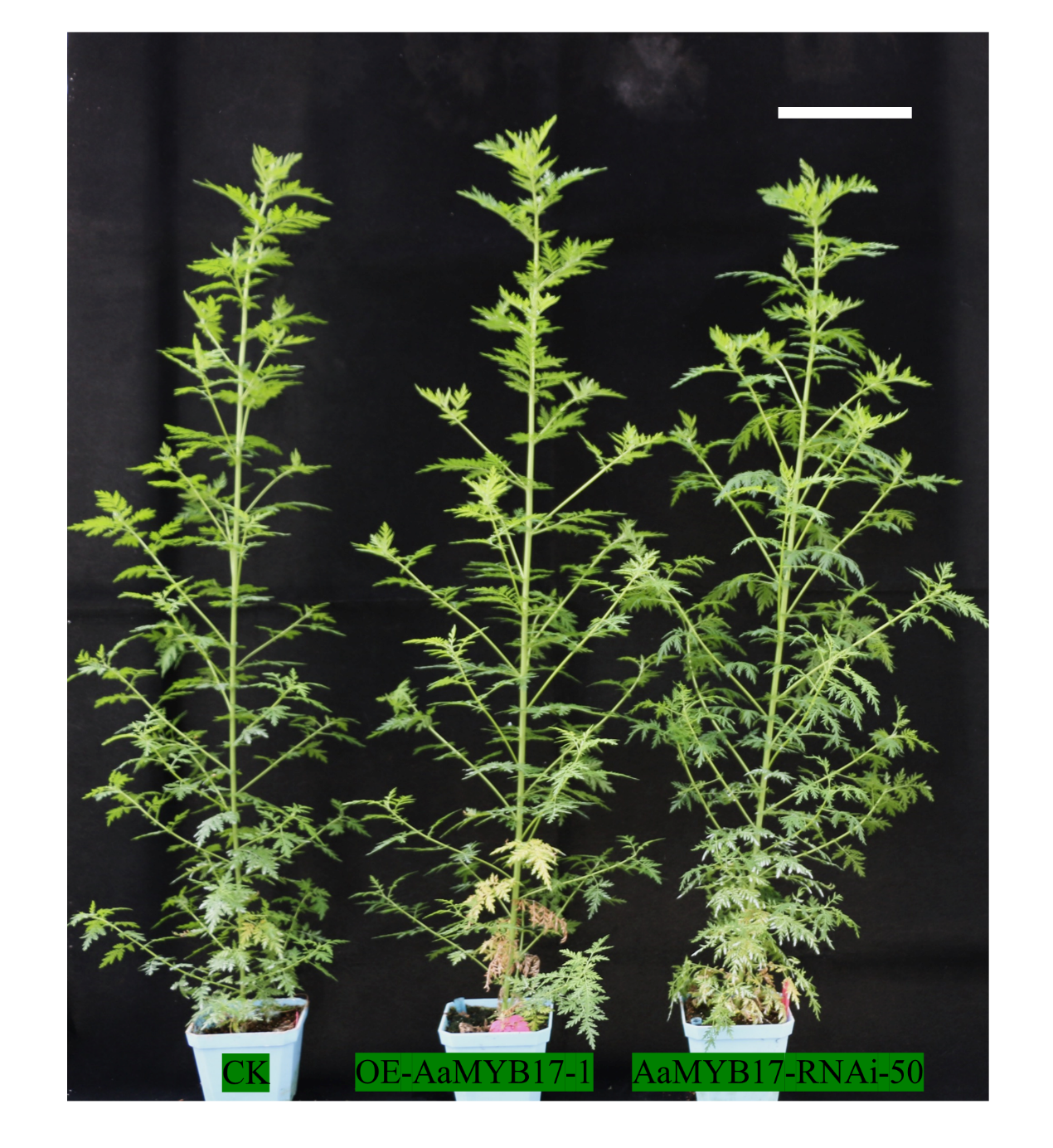


Figure. S2: Growth of *AaMYB17* transgenic plants. Bar:10 cm


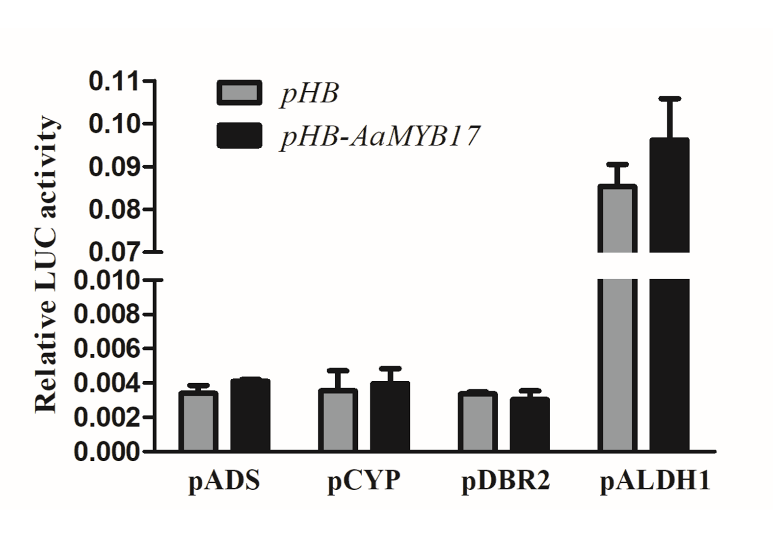


Figure. S3: Transient dual‐luciferase (Dual‐LUC) assay in *N. benthamiana* leaves. The values were calculated by the ratio of firefly luciferase activities to renilla luciferase activities (LUC/REN). Data are given as means ± SD (n = 3) (Student’s t-test, P > 0.05.)


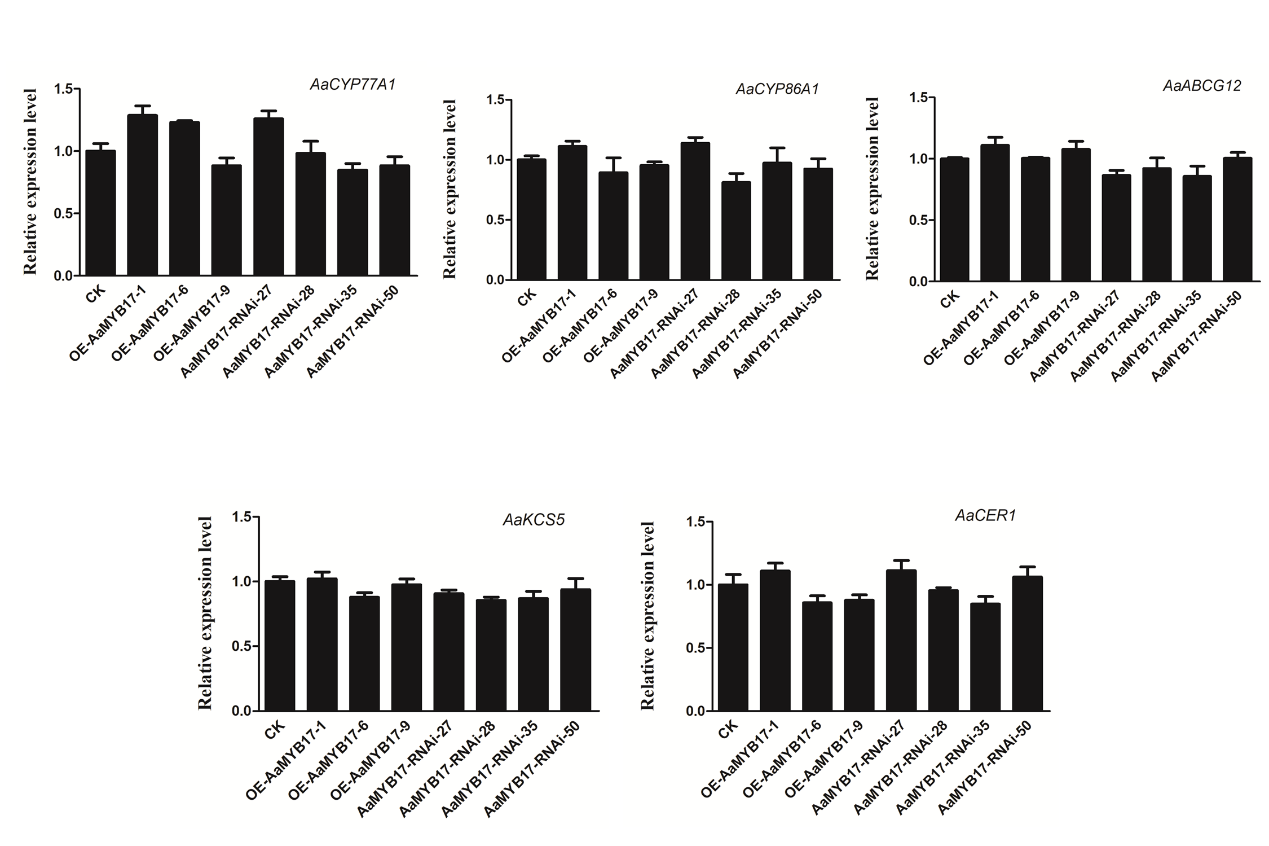


Figure.S4: Relative expression of genes involved in the cuticle biosynthesis in empty vector and *AaMYB17* transgenic *A. annua* lines by quantitative real‐time polymerase chain reaction. Three technical replicates were used for each biological repeat. Three biological repeats are measured for each sample. Error bars represented the standard deviation (n = 3).


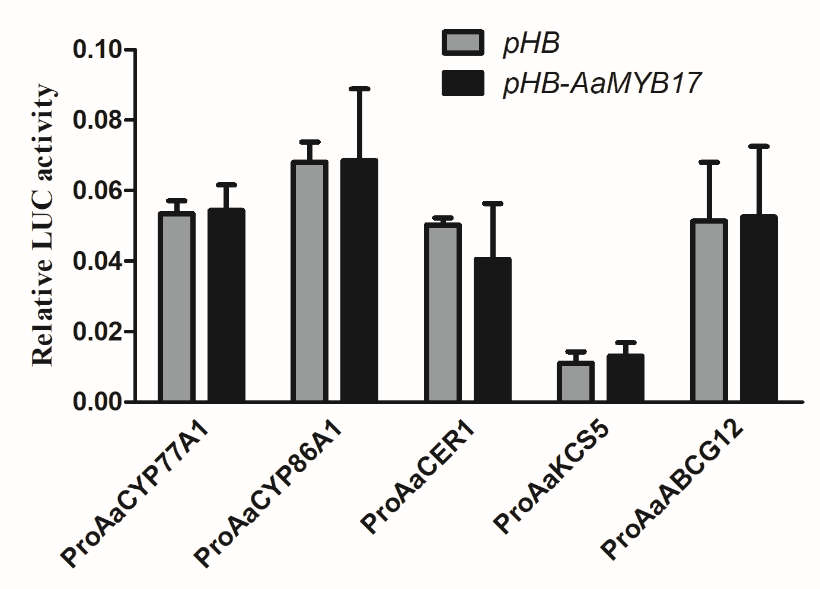


Figure. S5: Transient dual‐luciferase (Dual‐LUC) assay in *N. benthamiana* leaves. The values were calculated by the ratio of firefly luciferase activities to renilla luciferase activities (LUC/REN). Data are given as means ± SD (n = 3) (Student’s t-test, P > 0.05.)

Table S1 Primers used in the study

| Primer | Purpose | Primer Sequence (5'→3') |
| --- | --- | --- |
| AaMYB17-F | Clone | 5'-ATGGTGAGAGCACCATGTTGTGAGA-3' |
| AaMYB17-R | Clone | 5'-CTATATTTTTCGGCCCATGGAAATC-3' |
| AaMYB17-RTF | Q-RT- PCR | 5'-TCATTACTACTCCCATCACCCAAC-3' |
| AaMYB17-RTR | Q-RT- PCR | 5'-CATCGCTCCTTCTGGCACAT-3' |
| pAaMYB17-F | Clone | 5'-TGTGATTGGTAGTTTTGATGATGGT-3' |
| pAaMYB17-R | Clone | 5'-GGTTTTGTCCAAGAAACTTAAACAG-3' |
| 1391pAaMYB17-BamHI-F | GUS | 5'-CAGGTCGACGGATCCTGTGATTGGTAGTTTTGATGATGGT-3' |
| 1391pAaMYB17-Ncol-R | GUS | 5'-TCAGATCTACCATGGGGTTTTGTCCAAGAAACTTAAACAG-3' |
| pHB-AaMYB17-YFP-BamHI-F | Subcellular localization, Overexpression transgenic plants | 5'-TCTAAGCTTGGATCCATGGTGAGAGCACCATGTTGTGAGA-3' |
| pHB-AaMYB17-YFP-SpeI-F | Subcellular localization, Overexpression transgenic plants | 5'-GCTCACCATACTAGTTATTTTTCGGCCCATGGAAATC-3' |
| AaMYB17-RNAi-F | RNAi transgenic plants | 5' -AGCAAAACGACTCCCCAC -3' |
| AaMYB17-RNAi-R | RNAi transgenic plants | 5' -CTGTTTTAGGCACCATTTTG -3' |
| Actin-QF | Q-RT-PCR | 5'-CCAGGCTGTTCAGTCTCTGTAT-3' |
| Actin-QR | Q-RT-PCR | 5'-CGCTCGGTAAGGATCTTCATCA-3' |
| AaCYP77A1-QRT-FP | Q-RT-PCR | 5'-CATTCAAGTGGGTCTCCGTTC-3' |
| AaCYP77A1-QRT-RP | Q-RT-PCR | 5'-TTCGATTAAGGAAGGTCCTAATT-3' |
| AaCYP86A1-QRT-FP | Q-RT-PCR | 5'-TTCCCAAGCTCTTTGCTCTTC-3' |
| AaCYP86A1-QRT-RP | Q-RT-PCR | 5'-GCCACCATAAACATGATAATA-3' |
| AaABCG12-QRT-FP | Q-RT-PCR | 5'-CCCGTCTTCTAAACGACAGTA-3' |
| AaABCG12-QRT-RP | Q-RT-PCR | 5'-AGCACACAAATGAATTGTTATAG-3' |
| AaKCS5-QRT-FP | Q-RT-PCR | 5'-TTCTTCATCCCCTTCAGTTGC-3' |
| AaKCS5-QRT-RP | Q-RT-PCR | 5'-GGAGTAGTTGGATTACAGCGGC-3' |
| AaCER1-QRT-FP | Q-RT-PCR | 5'-GCCTGTCACAATCTCGTCCAT-3' |
| AaCER1-QRT-RP | Q-RT-PCR | 5'-TGCTAAACTACTCCCATCCACC-3' |
